# Supplementary figures and images for: A brighter future? Stable and growing sea turtle populations in the Republic of Maldives
Source: PLoS One. 2023 Apr 26;18(4):e0283973. doi: 10.1371/journal.pone.0283973 (PMC10132635; doi:10.1371/journal.pone.0283973)

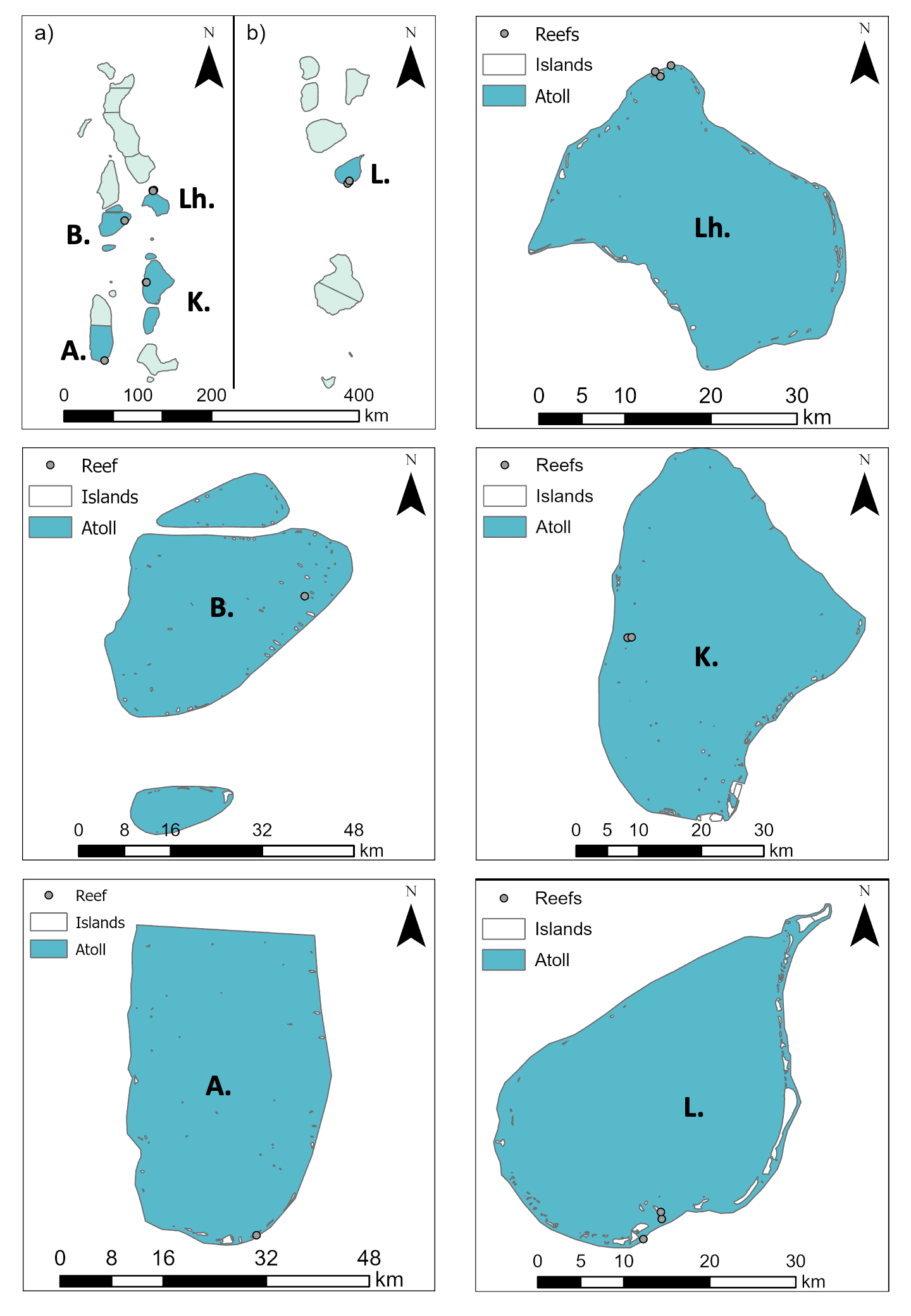

Supplement: S1 Fig — (TIF) [file pone.0283973.s001.tif]

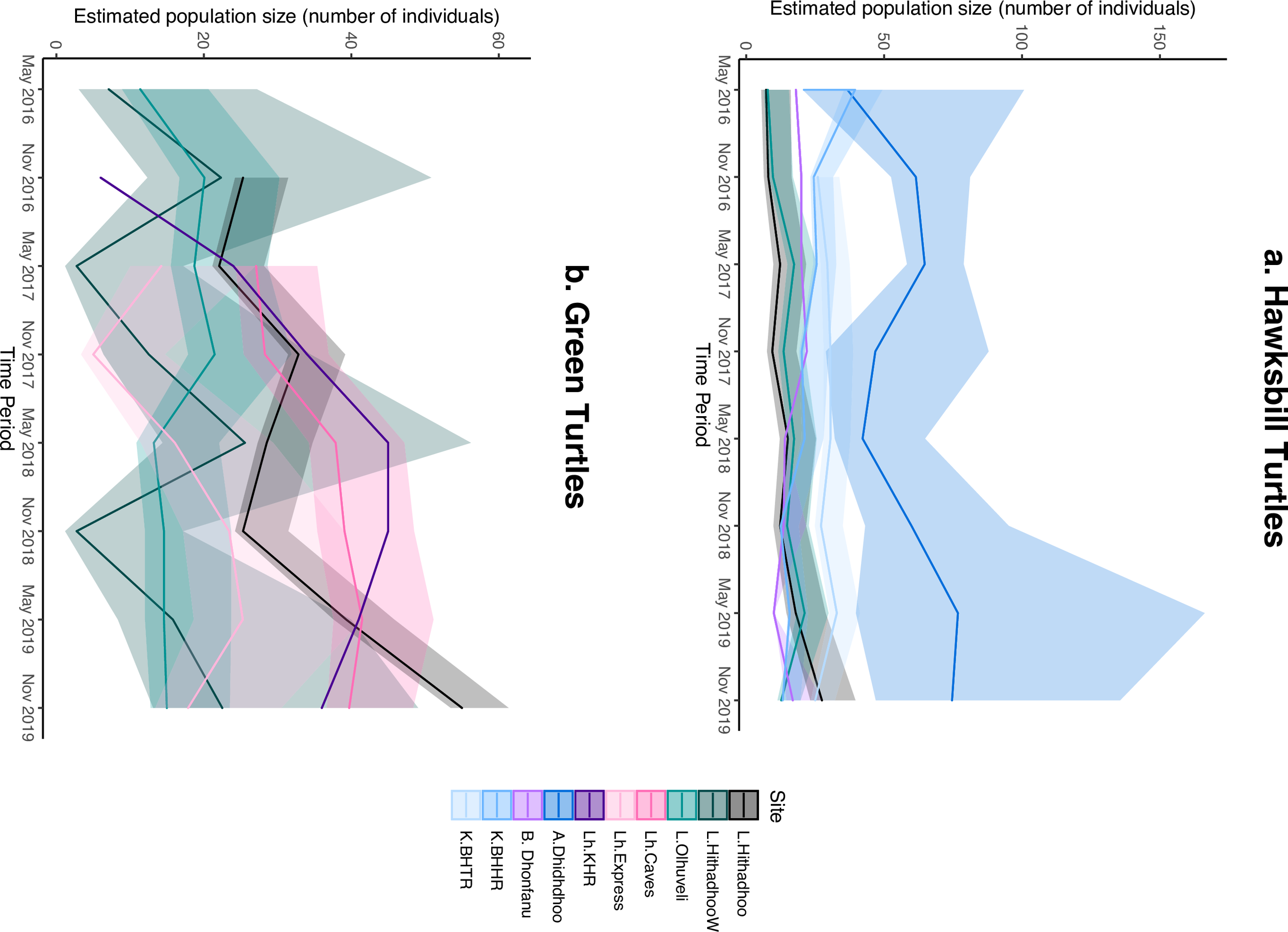

Supplement: S2 Fig — Population estimates with standard error shown at each primary interval for a. hawksbill b. green turtles. See S1 Table for a description of site codes. (TIF) [file pone.0283973.s002.tif]
