# Supplementary material for: A brighter future? Stable and growing sea turtle populations in the Republic of Maldives
Source: PLoS One. 2023 Apr 26;18(4):e0283973. doi: 10.1371/journal.pone.0283973 (PMC10132635; doi:10.1371/journal.pone.0283973)
Supplement: S1 Appendix — (DOCX) [file pone.0283973.s006.docx]

**Appendix 1.** Details of models tested.

The following three temporary emigration patterns were considered: Scenario 1: no temporary emigration; Scenario 2: random temporary emigration, where the probability of an individual being present in the study area is not dependent on whether or not it was present in the study area in the previous sampling period; and Scenario 3: Markovian temporary emigration, where the probability of an individual being present in the study area is conditional on whether it was present in the study area before (S3 Table). Standard open models result in biased estimates if there is Markovian temporary emigration [45]. Markovian emigration may be an early warning sign of population decline and it signifies that it is important to keep turtles on their home reefs and reduce disturbances. Therefore, the capacity for RD to account for temporary emigration is useful when estimating abundance in species that move in and out of a study area. For all three patterns of temporary emigration, we considered models where apparent survival was either constant or varying between primary periods and capture probability was either constant or varying with time (between secondary occasions, between primary periods, or both). We used the Akaike Information Criterion (AIC) to evaluate model fit [74]. The best fitting model was identified as having the lowest AIC [75].
