# Supplementary material for: A brighter future? Stable and growing sea turtle populations in the Republic of Maldives
Source: PLoS One. 2023 Apr 26;18(4):e0283973. doi: 10.1371/journal.pone.0283973 (PMC10132635; doi:10.1371/journal.pone.0283973)
Supplement: Attachment — Submitted filename: Reviewer Response Document.docx [file pone.0283973.s007.docx]

Response to reviewers

**Editor**

Dear Dr. Hudgins,

Thank you for submitting your manuscript to PLOS ONE. After careful consideration, we feel that it has merit but does not fully meet PLOS ONE’s publication criteria as it currently stands. Therefore, we invite you to submit a revised version of the manuscript that addresses the points raised during the review process.

Please follow the referee suggestions for how you need to rewrite this manuscript so that the writing is not so parochial and has broader appeal.

**Response:** We thank the editor and both reviewers for their helpful comments. We have taken each into careful consideration and have made substantial changes to the structure and writing of the manuscript. The manuscript is now worded in more general terms and in a more typical scientific article style.

Please include the following items when submitting your revised manuscript:

- A rebuttal letter that responds to each point raised by the academic editor and reviewer(s). You should upload this letter as a separate file labeled 'Response to Reviewers'.
- A marked-up copy of your manuscript that highlights changes made to the original version. You should upload this as a separate file labeled 'Revised Manuscript with Track Changes'.
- An unmarked version of your revised paper without tracked changes. You should upload this as a separate file labeled 'Manuscript'.

If you would like to make changes to your financial disclosure, please include your updated statement in your cover letter. Guidelines for resubmitting your figure files

1. Please ensure that your manuscript meets PLOS ONE's style requirements, including those for file naming. The PLOS ONE style templates can be found at

<https://journals.plos.org/plosone/s/file?id=wjVg/PLOSOne_formatting_sample_main_body.pdf> and

<https://journals.plos.org/plosone/s/file?id=ba62/PLOSOne_formatting_sample_title_authors_affiliations.pdf>

2. Please include a complete copy of PLOS’ questionnaire on inclusivity in global research in your revised manuscript. Our policy for research in this area aims to improve transparency in the reporting of research performed outside of researchers’ own country or community. The policy applies to researchers who have travelled to a different country to conduct research, research with Indigenous populations or their lands, and research on cultural artefacts. The questionnaire can also be requested at the journal’s discretion for any other submissions, even if these conditions are not met. Please find more information on the policy and a link to download a blank copy of the questionnaire here: <https://journals.plos.org/plosone/s/best-practices-in-research-reporting>. Please upload a completed version of your questionnaire as Supporting Information when you resubmit your manuscript.

3. We note that you have stated that you will provide repository information for your data at acceptance. Should your manuscript be accepted for publication, we will hold it until you provide the relevant accession numbers or DOIs necessary to access your data. If you wish to make changes to your Data Availability statement, please describe these changes in your cover letter and we will update your Data Availability statement to reflect the information you provide.

4. Please include captions for your Supporting Information files at the end of your manuscript, and update any in-text citations to match accordingly. Please see our Supporting Information guidelines for more information: <http://journals.plos.org/plosone/s/supporting-information>.

**Reviewer #1:**

Here photo id is used to estimate numbers of hawksbill and green turtles at foraging sites in the Maldives. Using mark-recapture approaches, estimates of survival and emigration are made. It is concluded that at many of the sites the numbers of turtles are stable or increasing.

This manuscript contains some interesting observations and nice analysis. The authors should be applauded for how they have collected an extensive photo-id database. While the data collection and analysis are nice, it is a shame that the manuscript is let down badly by the writing. At the moment the Discussion is poor, being almost entirely very parochial writing or repetition of the Results. So almost the entire Discussion need to be rewritten. I appreciate the authors may be new to academic writing, so I have tried to provide helpful comments to guide them about how to put together a Discussion. So a major revision is needed and the authors need to take great care placing their work into a much broader context. If the authors can make these improvements, then their work will be far more widely read and have much greater impact. Throughout the authors need to identify their key results and the discuss those results in the context of the broader literature. E.g. “Our findings that X,Y,Z occurred is similar/contrasts to what has been found in other studies around the world. For example in study A it was found X, while we showed Y …”

Response: We thank the reviewer for their comprehensive writing suggestions. Our author team includes both academics and practitioners, which may have impacted the writing style. We believe there is more than one way to present an effective academic article, and believe many of the readers of this article will be practitioners themselves, so they are important to keep in mind during the writing process. Nevertheless, we have substantially reworked the majority of the manuscript, paying particular attention to the discussion. At the same time, we have also made some independent stylistic decisions and did not adhere entirely to reviewer-suggested rewrites throughout.

Throughout it is frustrating that the authors have not included line numbers or even page numbers. I am surprised the manuscript was allowed to go to review in this state.

Response: We apologize for this oversight as we were expecting automatic line-numbering to occur during the manuscript submission process as is the case with some journals. We have taken care to include our own line numbering in this revision and have made reference to relevant line numbers in the updated manuscript in our responses. See the clean version (no tracked changes) for appropriate line numbers.

1. Abstract. “… mean apparent survival estimate of 82.5%±2.6% …”

There needs to be an element of TIME in a survival estimate. You should make your values estimates of annual survival so you can place in the context of wider values. See for example, Fig 2a in: “Schofield G et al. (2020). Long-term photo-id and satellite tracking reveal sex-biased survival linked to movements in an endangered species. Ecology 101, e03027. <https://doi.org/10.1002/ecy.3027>”

Response: We thank the reviewer for their useful suggestion. We note that our previous survival estimates were calculated per 6-month period. We have converted all of our survival estimates to annual percent survival values and now report them as such throughout the manuscript (e.g., lines 327-332).

2. Abstract. “… concurrent with recent studies on nesting.”

Delete. Nothing is presented on nesting.

Response: This has been deleted.

3. Introduction. “The lack of information on adult males is now worrying, due to the concerns of hatchling sex ratios that are becoming more heavily female-biased due to increasing temperatures at nesting beaches worldwide (24; 25).”

You set this up in the Introduction but never return to this topic in your Discussion. What were your adult sex ratios for each species ? Discuss your findings. There are reasons males might not be as rare as previously thought – see “A review of how the biology of male sea turtles may help mitigate female‑biased hatchling sex ratio skews in a warming climate. Marine Biology 169: 89. <https://doi.org/10.1007/s00227-022-04074-3>”. Did you find balanced adult sex ratios ? Elsewhere in the region, fairly balanced hatchling sex ratios have been estimated due to shading of nests and rainfall. See: “Male hatchling production in sea turtles from one of the world’s largest marine protected areas, the Chagos Archipelago. Scientific Reports, 6:20339. <http://dx.doi.org/10.1038/srep20339.>”. So skewed sex ratio might not be such a big problem in the Indian ocean. Discuss your findings in this context.

Response: We thank the Reviewer for the helpful suggestion and have included information on observed sex ratios in the results section (lines 265-269), as well as associated caveats in the Discussion (Study limitations paragraph, lines 471-480).

4. Introduction. Fidelity.

“Many previous studies have revealed the high site fidelity of both species (29; 30; 21; 31; 32).”

For a review of green turtles, see: “Shimada (2022). Fidelity to foraging sites after long migrations. Journal of Animal Ecology 89, 1008–1016. <https://doi.org/10.1111/1365-2656.13157.>”. Here you need to DISCUSS your findings in the context of the broader literature. What do your findings add ? When your turtles leave, do they subsequently return or do they leave for distant areas – see both of these scenarios in long-term tracking of hawksbills in the region: “High accuracy tracking reveals how small conservation areas can protect marine megafauna. Ecological Applications, 31(7), e02418. <https://doi.org/10.1002/eap.2418>”.

Or are they dying and so being removed from the population ? All this needs to be discussed in the context of the broader literature.

Response: We included the suggested review in this section and in the reference list (lines 764-765). Additionally, we included a paragraph on site fidelity in the Discussion (line 421-432).

5. Table 1. Not needed in main text. Put in SI.

Response: This table has been moved to the supplementary material section (now Table S1).

6. Life stages. Methods How were adult identified ? e.g. how did you distinguish a large sub-adult from an adult ? So presumably you estimated SIZE of turtles. Detail how this was all done. Be honest – if you are not really sure of adults V large sub-adults then say this. Turtles have been sized by divers previously in the region: “Houghton et al. (2003). Habitat utilisation of juvenile hawksbill turtles (Eretmochelys imbricata) in a shallow water coral reef habitat. Journal of Natural History 37, 1269-1280.”. Discuss your approach (in the Discussion), i.e. how good are your life-stage and size estimates ?

Response: We included a description of life stage and size estimates in the Methods section (lines 132-134) and added a discussion of our findings to our discussion section (lines 397-400; 421-432).

The following includes results you then need to discuss (in the discussion) in the context of the broader literature:

7. Life stages. What was the adult sex ratio ? Place in the context of the broader literature.

Response: We have added this in the Results as well as in the Discussion (see Response 3).

8. Where do the adults come from ? Place in the broader context. For example, satellite tracking has shown that some nesting green turtles from the Chagos Archipelago migrate to foraging grounds in the Maldives. See Figure 1 in: “Open ocean reorientation and challenges of island finding by sea turtles during long-distance migration. Current Biology 30, 3236–3242. <https://doi.org/10.1016/j.cub.2020.05.086>”. So some of the connections are known. Discuss. In contrast no tracked nesting hawksbill turtles from the Chagos Archipelago have travelled to the Maldives (see: “Travel routes to remote ocean targets reveal the map sense resolution for a marine migrant. Journal of the Royal Society Interface 20210859. [https://doi.org/10.1098/rsif.2021.0859”](https://doi.org/10.1098/rsif.2021.0859%E2%80%9D)), suggesting the adult hawksbills you see might be more locally derived ? Discuss.

Response: We thank the Reviewer for the helpful suggestion and have included a new paragraph on source locations, linking with existing literature, in the Discussion (lines 451-466).

9. Inferred emigration. Place in the broader context. For example long term tracking has shown protracted residence of immature hawksbill turtles in localised areas in the Chagos Archipelago while only a few individuals relocate. See: “High accuracy tracking reveals how small conservation areas can protect marine megafauna. Ecological Applications, 31(7), e02418. <https://doi.org/10.1002/eap.2418>”. See references within that paper for the broader literature from other sites. Place your observations into that wider context.

Response: We thank the Reviewer for the helpful suggestion and have now linked our inferred emigration patterns to the broader literature, including the suggested reference, in the Discussion (lines 409-419; 451-466).

10. “For hawksbills, apparent survival ranged from a low of 76% at …”

Place these findings into the broader context. For example, survival rates have been measured by photo id and other mark recapture approaches for a range of species. E.g. as a starting point see: “Long-term photo-id and satellite tracking reveal sex-biased survival linked to movements in an endangered species. Ecology 101, e03027. <https://doi.org/10.1002/ecy.3027>”. Discuss your findings within this broader context.

Response: We thank the Reviewer for the helpful suggestion and have now linked our apparent survival rates to the broader literature, including the suggested reference, in the Discussion (lines 382-391; 393-395).

11. “… the highest growth rates but …”

This reads as you were recording the growth rate of individuals. Rephrase. Here I think you are talking about an increase in abundance. Rephrase throughout rather than talking about “growth”.

Response: We have rephrased throughout the manuscript to indicate that we are referring to an increase in population abundance, and have specified 'population growth rate' in the equation representation and where rates needed to be reported/discussed.

12. Figure. “Estimated survival (%)” This is a strange unit. Survival needs to be calculated in % surviving per year (annual survival rate) and placed into a broader context. So extrapolate you measurement interval up to 365 days to get annual survival values.

Response: We thank the reviewer for their helpful suggestion and have recalculated and modified all reported survival values so that they are displayed as annual rates, rather than 6-month interval values.

13. What are trends in numbers of these species elsewhere in the region. E.g. as a starting point see Mortimer et al. Estimates of sea turtle nesting populations in the south-western Indian Ocean indicate the importance of the Chagos Archipelago. Oryx 54, 332–343. <https://doi.org/10.1017/S0030605319001108>. E.g. are encouraging upward or stable trends seen elsewhere in the Indian Ocean for hawksbills and greens. Place your results in this broader context. Are your finding consistent or different to what other have recently reported ?

Response: We have included a paragraph discussing our findings in comparison to other reported population trends as well as the connection between Maldivian and other Indian Ocean populations in the Discussion (lines 434-449), including the suggested reference.

14.. Discussion. “Hawksbills in the Arabian Gulf have been documented to migrate …”

This discussion needs to be expanded. What have others reported ? e.g. “Ecological Applications, 31(7), e02418. <https://doi.org/10.1002/eap.2418>”.

Response: We thank the reviewer for their helpful suggestion and have expanded the discussion to include further reference to site fidelity, potential reproductive migration, and other life history events of sea turtles (lines 365-380; 451-457).

The following are long sections of the Discussion that can be deleted as they are just background information or repetition. Instead build you discussion around discussing your results in the context of the broader literature.

15. Discussion. Repetition of Results. Delete: “We also hypothesized that estimated hawksbill abundance and stability would be higher than green turtle abundance and stability. This held largely true, indicating a greater relative need to protect green turtles, though we note that green turtle populations are estimated to be growing at all sites, while several sites had shrinking hawksbill populations.”

Response: We thank the reviewer for their helpful comments and have removed this section from the manuscript

16. Discussion. Repetition of Results. Delete:

“The hawksbill population at L.Hithadhoo had a high multiyear growth rate and relatively low CV, indicating it was home to a fast growing, stable population of hawksbill turtles. This site is best described by a model with no temporary emigration, indicating that turtles arrive at this reef and remain there. The populations at A.Dhidhdhoo and L.Olhuveli were also growing relatively quickly but their CVs were higher, indicating more instability in the populations at these sites. Both L.Olhuveli and A.Dhidhdhoo fit a model with no temporary emigration, though modeling for A.Dhidhdhoo showed temporal variation in detection probability across all turtles. Populations at K.BHTR and K.BHHR showed negative growth rates but relatively low CVs, indicating shrinking but relatively stable populations at these reefs. B.Dhonfanu also showed a negative growth rate along with a high CV, indicating an unstable, shrinking population. In addition, our results indicated a transient association with this reef. This could indicate that the turtles were leaving the reef due to disturbance.”

Response: We thank the reviewer for their helpful comments and have removed this section from the manuscript

17. Discussion. Very parochial and repetitive of Results. Delete: “The Dreaming of Maldives resort repository (dreamingofmaldives.com) indicates that several new resorts have been built in the Maldives in the last few years, particularly within Baa atoll, which would have caused noise and sedimentation during construction, as well as increased boat traffic and tourist presence. These additional stressors may have caused the unstable and transient nature of the turtle population at this site. Out of the six reefs analyzed here, one reef had a fast-growing stable population, two had stable shrinking populations, two had growing but unstable populations, and one reef had an unstable shrinking population. The overall development of the population of the country is still not entirely resolved. The mixed results from the currently investigated reefs could indicate a shift in habitat use, with turtles moving from sites with higher disturbances to other reefs. Currently, no such movement has been documented with the photo ID method, but this could be due to the continued under- sampling of reefs in the country.”

Response: We thank the reviewer for their helpful comments and have removed this section from the manuscript

18. Discussion. Repetition of Results. Delete. “Green Sea Turtles

Lh.KHR had the highest growth rate but a relatively high CV, indicating that the population was growing quickly but was relatively unstable. In addition, Lh.KHR fit a model of no emigration, but individual variation in detection probability, potentially indicating lower detectability at this site. There was high recruitment to Lh.KHR, but some turtles appear more challenging to relocate. Lh.KHR features extensive seagrass meadows, which provide an excellent foraging area for green turtles. It is possible that the observed population trends are an indicator for the function of this reef as a recruitment area and first stepping stone for greens coming into the archipelago. In total, the area has a large carrying capacity, but greens occasionally move on after some time. The difference in detection probability potentially resulted from individual variation in which area of the seagrass bed was used by the turtles. The first section of this site the lagoon closest to the jetty and main activity area is frequented more often than the second section further down the beach, resulting in different use by citizen scientists (i.e., tourists). L.HithadhooW and L.Hithadhoo both had relatively high growth rates, indicating that many turtles are immigrating to these sites. Both sites also fit models with no emigration, indicating strong retention of individuals across seasons. However, L.Hithadhoo displayed more variable detection, indicating potentially more sporadic surveying, while the former may display more unstable recruitment dynamics.”

Response: We have removed this section from the manuscript

19. Discussion. Repetition of Results. Delete. “L.Olhuveli had a low growth rate but a relatively high CV. It also had the lowest apparent survival of the reefs analyzed, indicating greater instability compared with other reefs, and a potential decline in habitat suitability over time. However, it fit a model with no emigration. There may have been some disturbance at this site, such as seagrass removal at nearby meadows that occurred up until 2018 (55). This could warrant further investigation, or it may indicate that carrying capacity of the reef (saturation) has been reached. Lh.Caves had a relatively low growth rate and higher CV, indicating that this population was moderately unstable but growing slowly. Finally, Lh.Express had the lowest growth rates but one of the lowest CVs, indicating that this population was stable and growing slowly. Modeling results also indicated that turtles were remaining at these reefs with strong fidelity.”

Response: We have removed this section from the manuscript

20. Repetition. Delete. “Out of the six reefs analyzed here, one reef had a fast-growing unstable population, one had an unstable slowly growing population, while the remaining four reefs were growing moderately and were moderately stable. However, we note that we only have 3.5 years of data over which we can ascertain trends, and longer-term datasets may reveal different growth and stability patterns.”

Response: We have removed this section from the manuscript

21. Background information. Not needed. Delete “Further Research

Determining population health of greens and hawksbill sea turtles without first defining their populations’ boundaries and management unit(s) (MU) comes with its challenges. Whilst our results are promising, we do not know how fragmented the MUs are. Future research should focus on uncovering the genetics of sea turtles in the country to better evaluate the impact of various threats. Moreover, improved phylogeography of green and hawksbill sea turtles in the Maldives will help resolve metapopulation dynamics in the region (56).”

Response: This section has been shortened as requested (lines xx-xx)

22. “Policy and management implications”

Delete this section. It is all background information and there is no discussion on your results in this context.

Response: We have removed this section from the manuscript

23. Conclusion. This long section is not needed. It repeats the Abstract. Reduce to 2 or 3 lines at most.

Response: This section has been shortened as requested (lines 507-518)

24. references. Inconsistent reference formatting. Take some care and put references in the correct style.

Response: We have now taken increased care to format references correctly, including newly added references.

25. No figure legends in the pdf.

Response: We apologize for this omission. We have merged multipanel figures into single panel PDF with informative titles. Please note that colours were used to keep site-level data consistent across figures.

26. Figure 2. Always include some units on figure. So “estimated population size (number of individuals)”

Response: This has been updated.

27. Figure 3. Needs to be annual survival rates. Current units are meaningless.

Response: Our previous survival estimates were for 6-month periods. As above, we have converted these values to annual survival.

28. Figure 3. Why are a and b needed ? Combine ? Hard to tell without a legend.

Response: These figures corresponded to different species. We now include figure captions in a section before pdf figures.

29 Figure 4. Growth rate should be “increase in estimate population size”. And units should not be “%” but need some element of time, e.g. % per year.

Response: We have updated our growth rate units to annual percent rates of increase

Reviewer #2: General

This is a really exciting paper, with some really useful insights on foraging sea turtles in a region with limited research. However, at present, this manuscript is set out as a report for a local management agency, rather than a broad scientific audience. I have made detailed comments throughout – do, look at my suggestions on the figures with the results combined. There is a lack of information on the robustness of the raw dataset and how issues in seasonal variation in data collection were overcome. The results are too detailed, with it being very difficult to interpret the key findings. The introduction and discussion also need broadening to draw on the existing literature on the general concepts covered in this study. I would very much like to see this work published, however, at present major revision is recommended.

Response: We thank the reviewer for their interest in our work and helpful suggestions for rewriting. As we mention in our response to reviewer 1, we have attempted to balance taking into account suggestions with allowing for our own stylistic choices, and to balance the suggestions from both reviewers, so we have not copied all suggestions for restructuring or rewriting to the letter. That said, we strongly believe that taking into account both reviewer’s suggestions has greatly improved this work. We've now removed many components of our results and moved them to supplementary materials. Our discussion now includes caveats on the quality of the raw dataset and associated limitations. We also now note the benefits of using the Robust Design methodology to control for seasonal variation in effort. We further describe rewriting of the discussion in responses below, and have made some modifications to the introduction (but see response 5)

Abstract

1. This is currently written as a report format, and needs a much clearer focus. First, what is the background reasoning for the study, i.e. is there limited data on foraging of sea turtles, and why is it of use to record recruitment and survival – in general, not specifically your site. What is the overarching aim – as you seem to have many, and then you add in an extra one regarding the tourists etc. There is a paper in itself just on how you standardise the ad hoc data before using it to infer survival etc, as resightings will be biased towards hotspots frequented by divers or tourists etc. You state how many records, but not how many unique turtles these represented of each species, and what range of unique individuals were seem across years - this is essential information that needs to be briefly presented to give context to the data.

Response: We are unclear on whether this was intended as a suggestion for the abstract or the paper as a whole, and have focused on providing these details within the main manuscript rather than crowd the abstract with methodological details. We do, however, now make the abstract more general in terms of study aims beyond the Maldives. See responses 2-4 for more specific changes made within the abstract.

2. There are too many results, and too much site specific information – what are the key trends – i.e. your overarching results should be stating whether you have similar numbers of greens and hawksbills, how do immigration, emigration and survival compare for greens and hawksbills compare – are there similarities/differences overall. The seasonal variation is very interesting, do both species exhibit this? The site specific idiosyncracies should be kept for the results/discussion, unless they have a profound impact on general trends.

Response: We have removed unnecessary results from the abstract and instead focus on seasonal variation and key differences among turtle species (lines 15-29).

3. The wider relevance of the study is unclear, you state that your study shows that the different sources of info are key, but you do not actually evaluate this or the effect of these different sources and potential bias, so I think this detracts from your findings.

Response: The ‘key’ component that these data provide is that they are the only information available in the Maldives on sea turtle populations. Therefore, they provide the only information that can be used to infer trends - despite their biases. We have attempted to clarify this within our introduction (lines 15-18, 26-29). We now highlight in our caveats section that we see this as a preliminary study that requires more systematic data collection to minimize error in the long term, but do not expect that within-site estimates would be biased in terms of relative trends and values, even if they were biased in terms of population estimates, so we do believe we can trust relative differences across species and seasons.

4. To me the key message is that turtles of both species exhibit seasonal use of your region for foraging – it would be interesting to see if this links to their respective food availabilities.

Response: Thank you for your suggestions. We have reworked the abstract to reflect changes made throughout the document in line with both reviewers’ comments and focus on the suggested key message.

Introduction

5. I would advise revising this entire section to something like

Paragraph 1 – why it is important to understand foraging behavior of wildlife – highlight seasonality of this – what drives this – climate, depletion of food resources etc… potentially increasing exposure to threats from human activities etc..

Paragraph 2 – issues in marine environment with monitoring immigration, emigration, survival, esp for sea turtles, with disparate foraging areas, seasonality of use, different behaviours adults/juveniles (many items to list here) – how various studies are overcoming this with different techniques (identify some), leading into photo id and citizen science, including issues

Paragraph 3 – sea turtles and foraging – different species forage on different substrate – draw on recent studies looking at foraging behaviour, patch to region use, how multiple species share foraging habitats (several great studies in Gulf of Mexico coming out)

Paragraph 4 – Study aim, objectives, hypotheses tested, wider anticipated importance

Again, this starts too turtle/region/site specific – I would advise changing the focus to seasonality of foraging by different wildlife, particularly herbivores and omnivores, this then leads into a paragraph on turtles and different foraging habits of different species etc. Many of the items drawn on in the introduction have broader relevance, but at present this is not captured.

Photo id – again, this is used widely for many wildlife, so again this can start far more broadly, with a clear explanation of the benefits. There are several recent studies drawing on citizen science to help build turtle databases, and other marine wildlife, so these should be integrated, along with potential issues in bias and how they are being overcome.

Response: We have now performed a substantial reworking of the introduction in line with both reviewer’s comments, without strictly using the structure suggested above. Our revised section ties much more deeply into existing literature as suggested by Reviewer 1 in the aim of expanding the scope of interest and impact of this article.

6. “Little historical data exist on marine turtle populations in the Maldives, which makes it difficult to study trends or evaluate the effectiveness of conservation measures put in place by the government” – be careful with this statement. I would advise deleting it, and instead focusing on what information has been published. At present, this paragraph is not set out as an introductory paragraph for a manuscript – this would fit better as an initial study region paragraph in the first part of the methods.

Response: We thank the reviewer for their helpful comments and have removed this section from the manuscript

Methods

7. “visibility is lower during the wet season and there are fewer tourists in the water.”

This is really important, as both issues can be driving variability in your observations.

Response: We have added a sentence that clarifies that these factors would reduce detectability of turtles in the wet season, which can be captured in a seasonal way and separated from factors linked to survival in our mode (lines xx-xx)l. We also note that our empirical findings do not align with the expected seasonal pattern of detection bias, and are actually opposed ot it, so we do not believe our results are due to seasonal changes in visibility.

8. “Sea turtles can be individually identified based on their arrangement of facial scales.” – this needs to be supported by source citations from the peer reviewed published literature.

Response: A reference was added to support this statement

9. “no harm to, or harassment of, turtles” – how can you confirm this, how close did people get to turtles to photograph facial scutes?

Response: Since we cannot definitively confirm this, we have removed 'harm and harassment' from the text. However, we note that all participants were given educational materials on best practices prior to beginning data collection. A training session was provided to data submitters. Code of Conduct materials, distributed to all data submitters can be found at: https://oliveridleyproject.org/wp-content/uploads/2019/07/Code-of-Conduct-Sea-Turtles-Olive-Ridley-Project.pdf

10. “We found large differences in survey effort around the country, and high turnover of staff at many locations, leading to data gaps and lack of data continuity” How did you address this issue in your models?

Response: We now clarify in text that these large differences in effort resulted in the restriction of which sites and time had sufficient data to be modelled (lines xx-xx). This is why we chose to only examine the sites we did over this brief period. Beyond this, differences in effort across sites are controlled for somewhat in the Robust Design model through the ability to account for changing probabilities of detection over time. We also reference several manuscripts subject to similar challenges in variable effort that use this method (22, 41-43). There are likely still some remaining biases in the data that would be ameliorated with the continuation of this monitoring program.

11. “it was approached slowly and as many photographs as possible were taken of the animal” How close?

Response: We cannot say for certain how close the turtles were approached, but all data contributors were informed of best practices beforehand, which recommended approach of not closer than 1.5m, and within 5m for photo ID collection. Training materials in the form of a Code of Conduct, as well as online training sessions were provided individually (see reviewer 2 response 9).

12. Photo database – who compiled this? Was it a single person or multiple people? What was the false positive/false negative rate? How were such issues overcome? Did you have some way of validating this process?

Response: The database was compiled by the study’s lead author. A sample of the entire database was checked by 2 independent verifiers via visual inspection (now mentioned lines 161-163. We did not keep track of the rate of false positives and false negatives within this verification process but note that the majority of the data have been through it. The software does not have a known false positive or false negative rate and was only used after 2019. This is the first usage of this software for turtle photo-ID, so sensitivity/specificity couldn’t be estimated. We provide the Internet of Turtles reference in text largely so that future data users can make use of it, rather than as a validation procedure.

13. “Incomplete data (missing identification, dates, or sites) were excluded from analysis” – more details are needed here; how much data did you start with, how many were removed? What % was retained?

Response: We have rephrased this to 'Only complete submissions were entered into the dataset.' (line 158-160)

14. In this study, six months (May to October and November to April) was – do you mean two six-month periods? This is not a six month period only, from what I can see.

Response: This sentence was rephrased for clarity as suggested.

15. For the model, first you need to clarify how bias and gaps were addressed/overcome.

Response: In our revised version, we now describe how we limited sites and study periods to the most consistent set of data, and from there how our model allows for differential detectability across time (lines 205-212).

16. Table 1 – this is not a main table, place it as online supplement.

Response: This table has been moved to the supplementary materials section (now Table S1).

17. Survival – you have a very short study period – can survival really be detected from this? Especially as your turtle numbers are very low at most sites.

Response: We now further highlight that the model predicts 'apparent survival' rather than survival, which includes departure from the study region. We do not aim to model mortality directly in this study (lines xx-xx). See also: <https://besjournals.onlinelibrary.wiley.com/doi/pdf/10.1111/2041-210X.12134>

Results

This is methods info:

18. We restricted site-level analyses to four atolls given the lower number of total turtles at the remaining atolls. Ten reefs across four atolls were chosen for analysis

Response: This section was moved to the methods section (lines 205-212).

19. Sentence beginning “In total, between 2016 and 2019 to end of paragraph” – the information the reader needs here is

1. How many records for each species and how many individuals overall

2. How were these split across the years – give mean and sd and range, so the reader can perceive the spread

3. How these were split by season – mean and sd and range for the two 6 month periods

4. How these were split by atoll – mean and sd overall, so the reader can perceive the spread – generate a table of the list you gave and refer to this for the individual atolls – these will have limited meaning to a general readership

Response: We added all of these additional pieces of information to the beginning of the results section (lines 240-257) and agree they provide a more complete picture of detection data that fed into model fitting (though it is important to keep in mind that these patterns have not been controlled for detection effort as in the model results, so patterns need not follow exactly). For point 2, we refrained from presenting the mean, sd and range and instead presented the counts for each year.

Life stages

20. This section is too wordy and too site specific. You have 6 atolls, I just need to know if there was an even spread in life stages or not for the two species. Present the detailed information as a graph and/or supplementary table. Capture the broad trends. If you want to keep site specific details, have them as online supplementary material.

Response: This section was shortened, as recommended, with site-specific details removed (lines 261-269).

21. Model interpretation, I would advise simply naming them reef 1 to 6, and clarifying which is which in supp table 1.

Response: The authors made the decision to keep the names of the reef rather than converting them to numbers in order to allow readers interested specifically in Maldivian sea turtle populations to have a deeper understanding of their demography. We see this as important, as ecological data are disproportionately less available from Small Island States.

22. This interpretation is very complicated. Again, it needs simplifying – what is the key overarching finding, what pattern did most reefs exhibit for each species compared to each other. There is some really interesting information, but it is lost in the detailed information. Place the details as online supplement and draw out the key points of interest for immigration/emigration and then survival – so there should be two clear simple sections.

Response: The authors have reworked the discussion section to clarify the results in terms of dominant trends across reefs and species (now lines 359-363; 365-380; 393-395; 409-419). The detailed results are available in the figures and supplementary tables (Fig.S2, Table S2).

23. Table 2 – make this an online supplement

Response: The table was included in the supplementary information section

24. Discussion – this needs to be revised. While the wider literature is drawn on, there is too much site specific information that detracts from the general broad findings of interest. This should not be separated into hawksbills and green turtles, rather paragraphs exploring emigration/immigration and survival trends as a whole, as well as overlap/segratation in foraging habitat use related to life stage, and the issues with photo id and citizen science encountered, including limitaitons..

Response: As suggested by the reviewer, we have restructured and extended the discussion to place all observations and findings into a broader context. See responses 25-30 for more detailed changes.

25. A paragraph on survival trends is needed and how your trends fit with the wider published literature – there are many studies that have explored this for adults and immature turtles, you need to build on this.

Response: A discussion of survival trends is now included in lines 382-291 and placed in the context of the literature.

I would advise 5 key paragraphs, i.e.

26. Para 1 – key overarching findings (these draw your key results together, and set the scene for the discursive content – comparing your findings to the wider literature in subsequent paragraphs)
27. Para 2 – distribution across atolls – similarity/difference across species, sex specific differences – overlap – segregation – how does this compare to other regions with mixed species – how does different foraging patterns affect this, how even was distribution
28. Para 3 – seasonality of habitat use – immigration/emigration how this compares to other locations/turtle species – what is driving it – monsoon, other factors, what drives it at other sites
29. Para 4 – effect of environment versus sampling effort – potential issues etc. Study limitations
30. Optional para 5 – management implications – keep it broad, relevant to wider audience (site specific stuff can go in an online supplement)

Para 5 conclusions

Responses 26-30: Key overarching findings are now discussed in lines 359-466 and placed in the context of existing literature.

The suggestion to perform more of an atoll-level comparison is interesting, but we would prefer not make any claims on differences between the two atolls based on the current database due to the small number of sites included. These sites were chosen due to their data availability, and are not necessarily representative of atoll-level differences. This is further evidenced by the fact that the data were too inconsistent across sites within an atoll to conduct a meaningful atoll-level RMARK analysis.

A discussion of seasonality, potential driving environmental factors, and immmigration/emigration is now included in lines 365-380; 409-419.

The previous section on model limitations was renamed "Study Limitations" and additional caveats of visual detection methods were added in line with previous reviewer comments. In spite of these limitations, we have highlighted the strengths of this work in its novelty for this region and for sea turtles at large. (lines 507-518

We have combined the suggested optional paragraph and conclusions in order to streamline the article.

31. Figure 1 – please change proportion to percentage. ON each bar, provide the n value, so it can be objectively compared.

What stands out to me here is that juveniles dominated for both, but that there were far more adult green turtles than hawksbills – indicating food availability is not sufficient for adult hawksbills

Response: Proportion was changed to percentage in this figure and we have highlighted the key results associated with it more clearly in the revised text (lines 261-269).

32. Figure 2 – What stands out here is one site is key for hawksbills and three for greens (with one key site for greens overlapping for hawksbills) – so, again ,what is of interest here is what is different at the key sites to the other sites for the two species – bathymetry, substrate, exposure? This is worth exploring.

Is the variation detected to do with variation in tourism/diving activity? Or is it truly a seasonal variation. If the latter, then greens exhibit more variability, which might be attributed to depletion of resources.

Response: We now highlight the key results more clearly in the revised texts. The reviewer’s suggested mechanism provides interesting ideas that could make for a strong future study, but cannot be answered within the scope of this study.

33. Figure 3 – I would be very wary of this graph, as your number of years of surveys is limited, your turtle numbers limited, and sampling effort inconsistent. I would advise acknowledging this in your results section – stating that survival ranged from x to x, but that sample sizes were low. So, when only focusing on the 1 and 3 key habitats of hawksbills and green turtles respectively. So change this to a single graph – it will be far more informative, and useful.

Response: We agree that our timescale is too short to draw meaningful implications on turtle survival or long term dispersal dynamics, but we do show important differences in the degree of persistence at each site. We have now weakened our language and added caveats to these findings, and highlight that our main focus is on determining key sites of association for each species (lines 327-332; 489-503). We prefer to show this as a multipanel figure to aid in the interpretation of each site studied for each species relative to one another, and note that uncertainties shown on this plot can help the reader understand the confidence in each result.

34. In the main text, it would be useful to state in this section what % of turtles were sighted every year (and once every 2 years, once every 3 years) – this then gives the reader an understanding of the robustness of your 5 year time series. i.e., if 90% of turtles are sighted every year, then fine. But, if 50% are sighted every year, and most return with 2-3 or 4 year intervals, then your 5 yr study timeframe is too short.

Response: Approximately 14% of hawksbills and 9% of greens were sighted every year. We now note this in text (line 243-245; 251-253). We are not aware of any heuristic for resighting frequencies and time series lengths required to perform an RD analysis. The resighting rate depends on many contextual factors (see Baldi et al. 2023 for an example of a much lower resighting rate for loggerhead turtles in spite of much longer timeseries). While we agree that longer timeseries would have helped minimize error in our model, the confidence intervals associated with our timeseries should be reflective in the uncertainty of our estimates. As we mention in the updated manuscript, even this preliminary study can provide important insights into demographic mechanisms and trends. We also further note the novelty of this analysis in our conclusions section (lines 507-518).

Baldi, G., Furii, G., Del Vecchio, M., Salvemini, P., Vallini, C., Angelini, V., ... & Casale, P. (2023). Behavioural plasticity in the use of a neritic foraging area by loggerhead sea turtles: insights from 37 years of capture–mark–recapture in the Adriatic Sea (Mediterranean Sea). *ICES Journal of Marine Science*, *80*(1), 210-217.

35. Also, how many individuals were recorded at more than one atoll within and across years?

Response: No turtles were sighted in more than one atoll, which has been added to the text (lines 402-403).

36. Figure missing – please give a map of the region and where the atolls are in relation to each other; this should also be stated in the main text, what is the minimum and maximum difference. Some atolls might indicate lower survival simply because resources were not present. If turtles move around a lot, then actually your data do not reflect survival but potentially use of many sites, with not all sites necessarily being viable in all years. On this figure, you should also identify other sites where turtles were detected foraging but were excluded to demonstrate the mosaic.

Response: This map has been added, though we decided against including specific datapoints for each turtle to avoid overwhelming the reader, as there were ~12,500 encounters in our study timeframe alone, over ~500 sites (now Figure S1).

Figure 4 – to me, 5 years is not long term, I am not sure this is viable; it depends on what the likelihood of resighting the same turtles is across years. Plus, this is simply driven by the 1 and 3 main sites for each species.

Response: We agree with the reviewer and had erroneously labelled this y-axis longterm instead of 'multiyear' growth rate. The Figure has since been corrected.
