# Supplementary material for: A brighter future? Stable and growing sea turtle populations in the Republic of Maldives
Source: PLoS One. 2023 Apr 26;18(4):e0283973. doi: 10.1371/journal.pone.0283973 (PMC10132635; doi:10.1371/journal.pone.0283973)
Supplement: Attachment — Submitted filename: Reviewer Response Round 2.docx [file pone.0283973.s008.docx]

**Editor’s comments:**

Your revised manuscript has now been evaluated by two of the original referees. You will see that the referees appreciate the improvements that have been made and they are generally happy with the revision. Nice work ! One referee suggests a few more minor changes. With a little care, I think you should be able to address all these remaining points and then this will make a very nice contribution to PLoS1.

Thanks again for all your hard work on the revision.

All best wishes, Graeme

Response: We thank the editor for their positive evaluation of our revised manuscript and further constructive comments below

**Reviewers’ Comments:**

Reviewer #1: The authors have made a good effort to revise the manuscript. Well done. There are still some things that I suggest you modify, avoiding repetition and clarifying the graphs.

1. Line 365-372.

Repetition of results delete.

Instead you can just say something like:

“Previous studies suggest temperature as well as benthic structure as important factors governing habitat use (51; 52; 53), which might explain the differences in abundance we recorded across atolls”.

Response: We thank the reviewer for their suggestion. Lines 365-372 were deleted and replaced with the suggested sentence.This section is now at lines 369-370.

2. Line 393-397. Repetition of Results. Delete.

Response: Lines 393-397 were deleted.

3. Lines 409-413. Delete this repletion of Results. “We detected emigration on three of the investigated sites for hawksbill turtles and on one for greens (see Table S2). At all but one of these four sites, random emigration was observed, indicating a constant rate of association and dissociation of turtles at these sites.”

Response: Lines 409-413 were simplified according to the reviewer’s suggestion.This section is now at lines 401-404.

4. On your graphs try and include a title and units, especially when presenting values. e.g. Figure 2a. Include a unit

Response: All graphs now have the proper units and titles (see also responses 5-6).

5. Figure 4. Think about the units of this figure. You say multiyear growth rate. This implies the growth of individuals. But I think this figure is about population size growth. So again, think carefully about your figures to avoid confusion.

Response: This graph now has a y-axis title of multiyear population growth rate.

6. Have a look at figures and decide which you think are clearest and follow them as a template. For example, x-y plots are typically wider than high. Shades of blue are perhaps not the clearest ?

Response: We thank the reviewer for their helpful suggestions for figure clarity. We originally chose the blue hues for this figure because they are colour-blind friendly, and did not want to re-use colours with the other figures where colour varies by site rather than life stage. We have now chosen a different colourblind-friendly scheme for Figure 1 that has a more stark contrast among values. We aimed to keep aspect ratios consistent across the other plots and have added units wherever needed.

7. Line 133. I think it is still not clear how you identified males V females and I am certain some of your sub-adults will be called “females”. You say:

“… those without a size estimate or clear identification as males based on tail length were recorded as sex and life stage unknown.”

But surely if they were not males, then you would call them females ?

Readers will be suspicious and if they are suspicious here, they might not lose faith with other aspects of your work. So I think it is far better to be honest and simply state something like:

“It is possible that females may have been wrongly identified as sub-adults and vice versa”.

Response: We thank the reviewer for their suggestion on how to clarify this point. Some additional language has been added to the sentence now starting on line 136. We have also added the statement suggested by the reviewer that some females could have indeed been incorrectly identified as we had no way of verifying the size estimates submitted.

Well done with making tremendous improvements to this manuscript. Graeme Hays

Response: Thank you very much for your constructive feedback that allowed for these improvements!

Reviewer #2: The authors have addressed the comments of the reviewers and it is greatly improved. Note, there are some typos and formatting issues in the discussion (minor).

Response: We thank the reviewer for their positive comments and have carefully checked the Discussion for typos and formatting inconsistency in the revised version.
